# Preventing suicidal behaviours with a multilevel intervention: a cluster randomised controlled trial

**DOI:** 10.1186/s12889-018-5032-6

**Published:** 2018-01-16

**Authors:** Sunny Collings, Gabrielle Jenkin, James Stanley, Sarah McKenzie, Simon Hatcher

**Affiliations:** 1 Suicide and Mental Health Research Group, University of Otago, PO Box 7343, Mein St, Newtown, Wellington, New Zealand; 2 University of Otago, PO Box 7343, Mein St, Newtown, Wellington, New Zealand; 3 Department of Psychiatry and Brain and Mind Research Institute, University of Ottawa, Ottawa, Canada

## Abstract

**Background:**

In the context of the recent surge in community based multilevel interventions for suicide prevention, all of which show promising results, we discuss the implications of the findings of such an intervention designed for and implemented in New Zealand. The multi-level intervention for suicide prevention in New Zealand (MISP-NZ) was a cluster randomised controlled community intervention trial involving eight hospital regions matched into four pairs and randomised to either the intervention or practice as usual (the control). Intervention regions received 25 months of interventions (01 June 2010 to 30 June 2012) including: 1) training in recognition of suicide risk factors; 2) workshops on mental health issues; 3) community based interventions (linking in with community events); and 4) distribution of print material and information on web-based resources.

**Results:**

There was no significant difference between the change in rate of suicidal behaviours (ISH or self-inflicted deaths) in the intervention group compared with the control group (rate ratio = 1.07, 95% CI 0.82, 1.38).

**Conclusions:**

This study did not provide substantive evidence that the MISP-NZ intervention had an effect on suicidal behaviours raising important questions about the potential effectiveness of the multilevel intervention model for suicide prevention for all countries. Although a range of factors may account for this unanticipated finding, including inadequate study power, differences in design and intervention focus, and country-specific contextual factors, it is possible that the effectiveness of the multilevel intervention model for reducing suicidal behaviours may have been overstated.

**Trial registration:**

This trial was retrospectively registered on 11 April 2013. ACTRN12613000399796.

## Introduction

Globally suicide is an enormous social and public health issue accounting for more than 800,000 deaths annually, with another 20 attempts for each suicide [1]. The World Health Organization has called for a comprehensive approach to suicide prevention with a global target of 10% reduction in suicide rates by 2020 [2].

Recently there has been a surge in community based multilevel intervention trials for suicide prevention. Multilevel intervention trials are distinct from multilevel interventions for suicide prevention occurring as part of national suicide prevention strategies, in that they are research based as exemplified by their intervention and control region research design. In this research context, multilevel intervention trials for suicide prevention have evolved in a particular way to include four or more common components. These four components have typically comprised: primary care interventions to improve the identification of depression, its treatment and management (including referral pathways); media/public relations campaigns to de-stigmatise depression and improve help-seeking; education of community gatekeepers; and interventions targeted to high risk groups (such as those with previous suicidal behaviours). Multilevel intervention trials have been designed for geographically defined communities, although some trials have been designed for specific population groups within a defined community (for example, senior citizens) [3].

Up to 17 such four-level intervention trials have been implemented in Europe [4]. It has been suggested that these multilevel interventions are the strategy of choice for community based suicide prevention [5–7]. Just how effective these initiatives are is of considerable interest because they are time consuming and resource intensive–hence costly. Multilevel intervention trials for suicide prevention typically involve the expert development of a study design (with intervention and control regions) and protocol, trial registration, complex ethical approvals processes and, once funded, staff recruitment, training and community engagement and participation over a long observation period. Despite a large number of multilevel intervention trials for suicide prevention being implemented, published results are only available for a small number. These results all claim to show a significant reduction in suicidal behaviours in the intervention versus control regions.

However, many more multilevel intervention trials have been implemented but not yet published. This may in part be due to the delay between trial completion and the publication of results (on average about 4 years). An alternative explanation, that of publication bias, where authors or publishers do not want to report non-significant or ‘negative’ findings, or delay their reporting, cannot be excluded [8].

In this paper we report on the results of our New Zealand community based multilevel intervention trial for suicide prevention: MISP-NZ. Contrary to the results of the previous published multilevel intervention trials, we found no significant reduction in suicidal behaviours after 2 years of intervention. This leads us to question whether multilevel interventions for suicide prevention are in fact the gold standard. We consider why our findings do not replicate those of similar trials and how such trials could be more effective.

## Background

### International context of suicide prevention

MISP-NZ was implemented in New Zealand between 01 June 2010 and 30 June 2012. Its design was informed by lessons learned from two prior multilevel suicide prevention intervention trials implemented in Akita, Japan [3] and Nuremberg, Germany and tailored to New Zealand [9]. The premise common to these two studies was that most mental disorders (including mood [10], substance use [11], anxiety [12] and personality disorders [13] [14]) were associated with an increased risk of suicidal behaviours, with psychological autopsy studies suggesting that the majority of people dying by suicide have a psychiatric disorder (typically depression), that was in the majority of cases, untreated [15, 16]. Thus, these trials (and a number recently), were designed on the premise that suicide rates could be reduced by introducing multilevel community intervention programmes that increase awareness of depression, its identification and management (especially in primary care), and available treatments [9].

### Multilevel suicide intervention studies informing MISP-NZ

#### Japan

Between 1985 and 2005, seven community based suicide prevention programmes lasting 5 years or more were implemented in Japan (a country with one of the highest suicide rates, at 25.5 per 100,00 population) [3]. These programmes all included depression screening and follow-up by physicians and the development of social networks in the community. Although the sample sizes were small, results indicated that such programmes would reduce suicide rates, at least in the rural areas where they were conducted [3].

#### Akita, Japan

One of these interventions was conducted between 1999 and 2004 in Akita, where the rates of suicide were highest of all 47 rural and urban prefectures in Japan [3, 17]. Using a nonrandomised quasi-experimental design, six small remote towns (total population in 2004 of 43,964) received 3 years of intervention and six towns in Akita were designated as controls (total population in 2004 of 297,071) [17]. The intervention and control towns both had a high proportion (more than 30%) of senior citizens (aged 65 or over). The Akita study interventions included: an awareness raising campaign for the general public (with the mid-life and older people the primary target population); public lectures (on the symptoms of depression and how to deal with it, the importance of listening to people with worries, and community based suicide prevention measures); public meetings; specialist training of welfare staff (in public health approaches to prevention of depression and suicide, and counselling skills); civic participation (in which community members planned their own activities such as awareness-raising lectures and theatrical performances); improved access to basic counselling services and voluntary mental health support workers; and a community network for older people (involving outings to share meals and engage in recreational activities). Rates of death by suicide were significantly lower in the intervention towns after the intervention at 34.1 per 100,000 population compared to the period before the intervention at 70.8 per 100,000 compared with the control towns (47.8 before and 49.1 after).

#### Nuremberg, Germany

The Nuremberg Alliance Against Depression [9] was a 2 year intervention (2001–2002) involving two German cities; Wurzburg, the control city (population 286,885) and Nuremberg, the intervention city (population 480,000), aiming to improve the care of depressed people and prevent suicidality. It consisted of four components: training of primary care physicians and support for the diagnosis and treatment of depression using educational packages; a media campaign (to improve mental health literacy about depression and reduce stigma) and work with local media on responsible reporting of suicide; training of community workers (including teachers, priests, counsellors and police); and a ‘green card’ scheme offering people in suicidal crises round the clock access to a specialist in suicidal crises. The study reported a statistically and clinically significant fall in the number of suicidal acts (defined as suicide attempts plus completed suicides), falling from 620 at baseline to 471 after the intervention – a reduction of 24% (mainly reduction in suicide attempts). For the control region the number of suicidal acts changed from 183 at baseline to 196 (7.1% increase) after the second intervention year [9]. This reduction in suicidal behaviour was sustained a year after the completion of the intervention [18].

There were a number of design limitations to both studies (the assignment of single cities as the control or intervention arm, and the lack of randomisation of cities to the study arm) but these two trials demonstrated it was feasible to implement complex multilevel suicide prevention interventions, and that these interventions appeared promising.

#### European alliance against depression

The apparent success of the Nuremberg study led to a European-wide (18 international partners representing 16 different European countries) intervention programme against depression and suicide, the European Alliance Against Depression (EAAD), funded by the European Commission [19]. Network countries have since been implementing and evaluating regional interventions comprising of four levels: training of general practitioners in the identification and treatment of depression and distribution of resources to educate patients and their families about depression; public awareness campaigns (to encourage treatment seeking by destigmatising depression and dispelling myths) and guidelines for journalists on safe reporting of suicide; interventions for high risk groups (for example, emergency card after a suicide attempt and providing aftercare resources and support through self-help groups), and 4) training sessions for community facilitators [20]. It has been claimed that the EAAD four level approach was recognised by the European Commission (in 2005) as a ‘best practice’ approach to reducing suicidality [19].

The EAAD approach was optimised and implemented in a 2008 joint project, Optimizing Suicide Prevention Interventions (OSPI), in four European model regions (Germany, Hungary, Ireland and Portugal) [4, 21]. OSPI-Europe was funded by the European Commission to provide health politicians, stakeholders and the European Commission with the evidence base (and corresponding materials) for efficient suicide prevention [21]. In OPSI-Europe the four-level model was complemented with efforts to restrict access to lethal means [4]. To date, only one of the four OSPI trials has been published: the trial conducted in Hungary.

In Hungary, between 2004 and 2005, Szolnok, a town of 76,311 (with one of the highest suicide rates in the world) received 2 years of interventions while Szeged, a town of 162,586, the control city, received practice as usual [22]. Results showed a significantly greater decrease in annual suicide rates (non-fatal attempts were not included) in the intervention region than the control (*p* = .0015) [22]. The annual suicide rate in Szolnok decreased from 30.1 per 100,000 in 2004 to 13.2 in 2005. For the control region the rate increased from 23.3 per 100,000 to 24.6 per 100,000 in 2005 [22]. Unlike the Nuremberg study, 2 and 3 years post intervention, the suicide rates in Szolnok returned to their higher pre-intervention levels, suggesting limited long term impact [22].

#### Recent evidence for the effectiveness of multilevel interventions

After MISP-NZ was designed, further evidence emerged adding to the accumulating support for depression focused multilevel intervention approaches for suicide prevention. Independently of the EAAD, a number of other countries have implemented multilevel suicide prevention interventions or action plans [7]. A synthesis of systematic reviews of multilevel suicide prevention interventions published up to 2011 concluded that there was evidence for the ‘actual or potential effectiveness’ of a number of components of multilevel suicide interventions. These were: training of GPs in the identification and treatment of mental disorders especially depression; public awareness campaigns (as long as there is a clear path to treatment); training of gatekeepers and community facilitators in recognising suicidality and helping at risk people access appropriate services; improvement to health services including making adequate aftercare and follow-up available for people who have attempted suicide; training of journalists in responsible reporting of suicide; and restricting access to lethal means of suicide [7].

Finally, there are the mixed results from a further multilevel intervention implemented in Japan. Based on the Akita study, suicide prevention interventions were delivered in rural and highly populated metropolitan areas from 2006 to 2009 [23]. As no effect was observed after 2 years of intervention, the study was extended to 3.5 years resulting in a reduced relative risk of suicide and suicide attempt by 7% in the rural areas (for men and elderly only), but no effect observed in the metropolitan areas.

### Misp-NZ

#### Objective

The objective was to determine whether the MISP-NZ interventions were more effective in reducing rates of suicidal behaviours (defined as intentional self-harm and self-inflicted deaths combined) in the four intervention regions than practice as usual (PAU) in the four control regions.

## Methods

### Study design

MISP-NZ was a cluster randomised controlled trial (cluster RCT) with randomisation to intervention/control arm applied at the level of the DHB. The intervention period ran for 25 months from 01 June 2010 to 30 June 2012 (pre-determined study duration). In the 6 months prior to this baseline data on intentional self harm (ISH)/self-inflicted death rates in both intervention and control DHBs was collected.

### Setting

New Zealand is a European-colonised, high income country, with a population of around 4.4 million spread mainly across its North and South Islands. Latest official suicide statistics for NZ reveal a rate of 10.7 per 100,000 population in 2014; a decrease from the peak rate of 15.1 deaths per 100,000 population in 1998 [24, 25]. In NZ, public health, primary care and hospital based care are delivered by 20 District Health Boards (DHBs). Each DHB is responsible for ensuring the public funds are directed to achieving government defined national level health priorities. About 80% of all health expenditure in New Zealand is from the public purse [26]. This is supported with voluntary private health insurance for those who can afford it.

### Eligibility and recruitment

The pool of 20 potential DHBs ranged in catchments from 31,000 to 481,000 people (population based on the 2006 NZ Census). Prior to randomisation, DHBs were matched on a variety of demographic factors (see below) and, from 20 DHBs in New Zealand, 4 pairs of DHBs (8 DHBs total) were selected for the study. Recruitment of the 8 DHBs took place prior to randomisation to the intervention and control arms – this meant that the DHBs agreed to take part with no knowledge of whether they would receive the intervention. Following randomisation, DHBs were no longer blinded to the study arm.

### Selection of matched pairs

Prior to randomisation, potential matchings of pairs of DHBs were assessed. The initial step included computer-handled matching on (1) age-standardised suicide rates for the 2002–2006 period [27]; (2) proportion of population falling in quintiles 4 or 5 of the New Zealand Deprivation Index (an eight item, area based measure of socioeconomic deprivation based on census data: in other words matching on the proportion of population who could be considered socioeconomically deprived); (3) DHB population size; (4) number of full-time-equivalent general practitioners. All these data were collated from the 2006 NZ Census (with the exception of suicide rates and number of general practitioners, as referenced above).

Tolerance levels were set for the initial matching process (e.g. rate ratio of suicide rates to be between 1 and 1.5) to produce a number of potentially well-matched pairs of DHBs. Four pairs of DHBs were then selected from this listing by members of the study team based on the pre-specified matching process, as well as less quantifiable factors or variables that were not explicitly included in the matching process (e.g. urban/rural profile of DHBs; scope of existing suicide prevention strategies in the DHBs). The selection of DHB pairs at this stage also took into account other pragmatic considerations (e.g. including geographically adjacent DHBs in the study was considered to be sub-optimal, especially if these DHBs had relatively contiguous urban areas).

### Randomisation

Randomisation within each DHB pair to the MISP-NZ intervention or practice as usual (PAU) arm was conducted using a random number generator process in SAS 9.1. This randomisation was performed by a biostatistician independent to the study team.

### Sample size calculation

A priori power calculations used a calculation method that accounted for pairing of DHBs [28]. The calculations drew on (1) current estimates of average intentional self-harm by DHB region, including annual variation in rates (150 per 100,000 person years); (2) between-DHB variability in annual ISH rates (standard deviation of 40 per 100,000 person years) in the absence of any intervention; (3) 2 years of follow-up data; and (4) a difference to detect of a 20% relative reduction in rates between intervention and control DHBs. Four DHB pairs yielded a power of 95.6% to detect a 20% reduction in rate in the intervention group, predicated on a summary-statistics comparison of outcome effectiveness (see primary analysis section) [28].

### Primary outcome suicidal behaviours

The primary outcome was the annual rate of suicidal behaviours defined as ISH resulting in presentation to the Emergency Department (ED) *and* self-inflicted deaths per 100,000 population, evaluated over the study period. ISH included all presentations of attempted suicide and self-harm. It was measured as the number of identified ISH events in the ED data (as the numerator) relative to DHB population sizes (as a person-time denominator). The process for identifying ISH used in the MISP-NZ study was more thorough than that used in the collection of national data on ISH and was determined collectively by the research assistants responsible for coding ED admissions after intensive examination of multiple medical forms and records. The person-time used in the denominator was derived from the entire population for the study DHBs from the 2006 New Zealand census (see Table 1), scaled for the duration of the baseline period (6 months) and intervention period (25 months). Counts of self-inflicted deaths, which included all completed (and suspected) suicides, were obtained from the Coronial Services of New Zealand. ISH was used as the primary marker of the intervention’s effect on suicidal behaviours because the number of self-inflicted deaths was likely to be too small to use as the primary outcome, which would have led to insufficient power to detect a difference of a clinically important magnitude.

**Table 1:** Primary outcomes (with 95% CI) by DHB pair, Study Arm and Time Period. Rates of intentional self-harm presentations to Emergency Departments/death by suicide; rate ratio for change in rate within each DHB by from pre-intervention to intervention period; rate ratio for difference in these changes in rate within each DHB pair

					Intentional self-harm/death by suicide				
DHB	Study Arm	Period*1	Events (deaths*2)	PYAR *3	Rate per 100,000 PYAR	Rate ratio for period*4		Rate ratio for intervention*5	
					(95% CI)		(95% CI)		(95% CI)
A	Intervention	Baseline	231 (13)	52,139	443 (389–504)	0.94	(0.81–1.08)	0.95 (0.87–1.26)	
		Intervention	902 (33)	217,244	415 (389–443)				
	Control	Baseline	392 (13)	79,422	494 (447–545)	0.98	(0.88–1.10)		
		Intervention	1605 (61)	330,925	485 (462–509)				
B	Intervention	Baseline	689 (11)	133,329	517 (480–557)	0.86	(0.79–0.94)	0.93 (0.94–1.23)	
		Intervention	2477 (53)	555,538	446 (429–464)				
	Control	Baseline	426 (21)	89,697	475 (432–522)	0.93	(0.83–1.03)		
		Intervention	1642 (68)	373,738	439 (419–461)				
C	Intervention	Baseline	342 (10)	97,467	351 (316–390)	0.92	(0.81–1.03)	1.09 (0.77–1.09)	
		Intervention	1305 (64)	406,113	321 (304–339)				
	Control	Baseline	325 (11)	74,124	438 (393–489)	0.84	(0.74–0.95)		
		Intervention	1135 (49)	308,850	367 (347–390)				
D	Intervention	Baseline	230 (6)	74,219	310 (272–353)	0.96	(0.83–1.10)	1.34 (0.63–0.87)	
		Intervention	916 (46)	309,244	296 (278–316)				
	Control	Baseline	925 (24)	216,542	427 (401–456)	0.71	(0.66–0.77)		
		Intervention	2739 (111)	902,256	304 (292–315)				

Footnotes for Table 1:

*1 Baseline period 6 months; Intervention period 25 months duration

*2 Events count is number of ISH events and suicide deaths combined (number in parentheses is number of deaths in period)

*3 PYAR = Person Years At Risk (based on population derived from 2006 Census, multiplied by duration of period)

*4 Rate ratio within each DHB for rate of outcome (intentional self-harm/death) during intervention period relative to baseline period

*5 Ratio in intervention arm (relative to control) within each DHB pair for the intervention to baseline ratio for change in outcome (intentional self-harm/death)

### Analysis

The total population within each DHB region was considered to be effectively exposed to the intervention. Primary analysis compared relative reductions in DHB-level ISH and self-inflicted death rates (reduction from baseline to intervention period) between intervention and control DHBs using a paired t-test (on the log of the ratio variable), to formally account for the matching of DHBs. This summary-measures approach to dealing with the clustered nature of the data accounted for the matched component of the study design [29]. Confidence intervals for these summary statistic estimates were calculated using the t-distribution. To summarise the potential impact of clustering, the coefficient of variation (k) was calculated based on the per-DHB baseline rate data pooled across study arms [30]. All DHB pairs were included in analyses of outcomes.

Additional analysis used a generalized linear model approach (PROC GLIMMIX in SAS 9.1) to compare the rates of ISH between the intervention and control DHBs, assuming a Poisson distribution for ISH events (arising from the count nature of the data) and accounting for the explicit pairing of DHBs. These results are summarised as rates of ISH presentation per 100,000 person-years at risk (PYAR), rate ratios for the difference between intervention and baseline periods within each individual DHB, and an “intervention effect ratio” calculated for each DHB pair that represents the relative difference in changes between each intervention DHB and its paired control DHB. These rates and rate ratios are presented with 95% confidence intervals. Additional analyses used the above approaches to look at outcomes restricted to (a) only ED presentation ISH events; and (b) only self-inflicted death events. No interim analyses were planned or conducted.

### Ethical approvals and consents

For the MISP-NZ trial (ACTRN12613000399796), ethical approval was obtained from the New Zealand Northern Y Ethics Committee after completion of the University of Otago’s Māori (Ngai Tahu) Research Consultation process and consultation with the DHBs involved. As a requirement of ethical approval, senior DHB staff signed an agreement granting access to Emergency Department data and providing permission for the delivery of the interventions in their DHB region.

### Organisation and data collection

The MISP-NZ team comprised of 21 personnel. The core project team of six, based at the University of Otago, Wellington, collectively managed the wider team’s involvement in MISP-NZ. The four intervention regions each had two clinical intervention staff (responsible for the development and delivery of the interventions). Each of the intervention and the control regions had a research assistant who collected ISH Emergency Department (ED) data for the primary outcome measure.

### Practice as usual (PAU) in the control regions

In New Zealand, aside from the Ministry of Health, the key national agencies engaged in suicide prevention are Te Pou (the National Centre for Mental Health Research) and the Mental Health Foundation. Te Pou works to support and develop the mental health workforce by providing tools and resources to improve mental health services (http://www.tepou.co.nz/). The Mental Health Foundation provides resources and advocacy on mental health issues, including the provision of high quality information to promote safe and effective suicide prevention activities (https://www.mentalhealth.org.nz/).

Two national programmes to improve awareness of mental illness in the community had been deployed prior to MISP-NZ. These were the Ministry of Health funded *Like Minds, Like Mine* programme [31] and the *National Depression Initiative*. *Like Minds, Like Mine,* is a public education programme aimed at reducing stigma (particularly internalised stigma) and discrimination associated with mental illness. The programme included national television advertising campaigns (with a free phone information service), communications and event management, provider education and training (for government and non-government community and health care organisations) and a website providing information and support on discrimination. Each DHB had one local *Like Minds, Like Mine* health and community provider whose role was to deliver a range of anti-discrimination activities including workshops, promotional events (raising public awareness of stigma and discrimination) and to assist in the development of non-discriminatory policies and procedures.

The *National Depression Initiative* (NDI) aims to reduce the impact of depression on the population, by aiding early recognition, appropriate treatment and recovery [32]. The NDI included television, radio and online advertising, and the provision and distribution of health resources; clinical care guidelines for primary care; phone, online and SMS text-based support services; and a website on depression. Another component of the NDI was a mental health and depression awareness campaign fronted by a national rugby celebrity, who has spoken openly about his battle with depression writing about it in his book *All Blacks Don’t Cry* [33]. This was supplemented with an online self-management programme for people with depression [34]. A separate website and online support service, the *Lowdown,* was available for young people [35].

#### Primary care

Primary care in New Zealand is a core function of the health system, being widely accessible as the first point of contact with the health system [36]. All but the poorest New Zealanders are required to pay or make a co-payment upon seeing their GP in primary care.

In 2003 the Ministry of Health provided the first ever funding for specific provision for primary mental health care (PMHC) under the Primary Mental Health Initiatives [37]. The target population was those with ‘mild to moderate’ mental disorders. The aims were to develop prevention, early intervention and treatment activities that would reduce the prevalence of common mental disorders such as depression and anxiety; develop PMHC workforce capacity and capability; and build effective links with other mental health care providers, especially but not solely secondary care, so that primary care could become an effective coordinator of care for people with enduring disorders. This provision was well embedded and ongoing during the MISP-NZ study.

### Interventions

In the eight DHB regions a high level assessment of existing mental health and suicide prevention activities and resources was conducted. Prior to the start of interventions. A detailed stocktake was undertaken in the four intervention DHBs. This enabled MISP-NZ staff to familiarise themselves with existing services, develop relationships with the service providers, identify potential gaps in services and develop appropriate local interventions. Drawing on the information collected in the stocktakes, and in consultation with service providers, multilevel interventions were designed for each region. Like the Nuremberg and other EAAD studies, an important focus for the MISP-NZ interventions was the primary care setting (interventions 1, 2, and 4 were delivered in this setting). However, unlike the EAAD trials which focused on the identification and treatment of depression, the MISP-NZ trial had a focus on identification of suicide risk (due to pre-existing investment in the national depression awareness campaign and development and use of depression identification and treatment guidelines for primary care).

#### Training in recognition of suicide risk factors

Lay and professional individuals working in the primary mental health sector, NGOs, community organisations were offered suicide prevention training. MISP-NZ utilised a New Zealand adaptation of Question, Persuade, Refer (QPR), an evidence-based suicide prevention training module accessible via the internet [38]. QPR provides simple evidence-based information about suicide, such as risk factors and key aspects of suicide prevention including identifying those at risk and how to encourage help seeking. MISP-NZ distributed 3008 modules of QPR in the intervention DHBs (uptake was 45% over all sectors and 47% amongst those working in primary care).

#### Workshops on mental health topics

In intervention DHBs, 317 workshops on topics relevant to the target groups and settings were designed and delivered. The topics were tailored to local needs (audience, setting and target groups) and included general suicide prevention, mental health awareness, alcohol and drug issues, anxiety, stress and self-harm. These workshops were hosted by 282 organisations across the range of health and non-health settings, including primary care practices, NGOs, local government, places of employment, local bank branches, prisons, courts, Māori organisations, church, social and family support groups. Individuals attending the workshops included health professionals, community workers and those from non-health backgrounds, the lay public and family members. Over all four intervention DHBs, the uptake of the workshops by organisations approached was 34% (uptake was lowest for small businesses). A separate process evaluation (unpublished) revealed various reasons for this low uptake. These workshops were the most resource intensive of the MISP-NZ interventions, requiring persistence, after hours availability, and cultural competence to successfully engage with Māori and Pacific groups. Factors facilitating agreement to hold workshops included, personal relevance, the provision of food to workshop participants as an enticement, pre-existing relationships with organisations and narrative fidelity (personal experience of the organisation’s contact person with suicide or mental health issues). Some groups felt pre-existing suicide prevention activities undertaken were adequate.

#### Community based interventions – Linking with community events

Eighty-one community based interventions were delivered. These typically involved local networking, advocacy and support and the provision of information resources at community events. Often these linked in with activities in local libraries and community centres as well as family days and other social events, such as mental health weeks and remembrance services, men’s working groups (e.g. in the building industry) and organisations. Additionally, MISP-NZ staff worked with local media to support best practice in suicide reporting. This included delivering workshops to media professionals on safe reporting and presentations by the Principal Investigator (SC), to print, TV and radio journalists. The media were supported to produce and disseminate educational materials on depression awareness and suicide prevention. Intervention staff also worked in with the radio and print media in their region to promote MISP-NZ activities, advertising workshops and upcoming events and promoting suicide prevention resources.

#### Distribution of print material and information on web-based resources

MISP-NZ distributed more than 89,000 suicide and mental health related print resources in the intervention DHBs. Many of these were leaflets and posters from pre-existing suicide prevention activities. Some new resources were developed targeted to at-risk population groups. For instance, business sized cards with the titles *‘Worried about a mate?’* and ‘RU OK?’ were developed for men, providing suggestions should they be concerned about a friend and information on where to go for help.

## Results

Crude rates for the composite outcome (ISH presentation to ED and self-inflicted death) during the six-month baseline period are shown in Table 1 (rate per 100,000 person-years at risk, with 95% CI) for DHBs in each of the four pairs. These rates varied across DHBs in the baseline period from 310 to 517 events per 100,000 PYAR, giving a coefficient of variation across clusters of k = 0.448. While DHB pairs had been selected based on national routine data on ISH, the measured baseline rates of ED presentations were somewhat discordant within the DHB pairs [39].

In the intervention period, crude rates of the composite ISH/self-inflicted death outcome ranged from 296 to 485 events per 100,000 PYAR (Table 1). Across all DHBs, the rate of ISH in the intervention period was nominally lower than the rate in the baseline period: as can be seen in the rate ratio for period column (Table 1) with considerable variation in this reduction between DHBs in each study arm.

Comparison of baseline and intervention rate ratios for the DHB pairs are given in Table 1 (with 95% CI). For all DHBs the point estimate of this reduction sat below the null value of one (which would indicate no difference in rates between periods): in three DHBs the confidence interval for the RR excluded one, while in the other five DHBs the confidence interval for the RR included this null value.

The primary outcome analysis compared the intervention period reduction in rates between the intervention and control arms, using a summary statistics analysis. In two of the four pairs of DHBs, the point estimate for this relative difference was lower for the intervention arm (Table 1); in the other two pairs, the point estimate for reduction was lower in the control arm. The intervention effect RR describes the relative differences in ISH rate reductions for the intervention arm relative to the control arm, giving an intervention effect RR of 1.07 (95% CI 0.82, 1.38). This indicates that ISH rates were 7% higher in the intervention arm when taking into account the relative reduction seen in the control arm (with the latter serving as a proxy for secular trends in ISH rates in the absence of this multilevel intervention).

Table 2 gives results from the generalized linear mixed models (GLMM) for rate comparisons, and gives the relative reduction from baseline to intervention for each study arm (rate ratio and 95% CI), as well as the relative difference in these reductions (intervention effect ratio.) The GLMM estimate of the intervention effect for the combined ISH presentation/death outcome was similar to that obtained from the summary statistic estimate calculated above (Table 2, for the combined ISH and self-inflicted death rates outcome).

**Table 2:** Rate ratios (95% CI) for reduction in suicidal behaviours between study periods in the intervention and control groups, with intervention effect ratio (reduction in intervention period in intervention arm relative to control arm)

	Rate ratio (95% CI^a^)		Intervention effect ratio^b^
	(intervention period relative to baseline)		(95% CI^a^)
Outcome variable	Intervention	Control	
ISH presentations and self-inflicted death events	0.91 (0.81–1.02)	0.85 (0.76–0.95)	1.07 (0.82–1.40)
ISH presentations only	0.91 (0.80–1.02)	0.85 (0.75–0.95)	1.07 (0.82–1.40)
Self-inflicted death events only	1.17 (0.84–1.65)	1.01 (0.77–1.31)	1.18 (0.51–2.70)

^a^95% confidence interval for estimate

^b^Intervention effect ratio is the between-period change in the intervention arm divided by the between-period change in the control arm. A ratio less than one indicates more favourable change in the intervention arm than the control arm; a ratio greater than one indicates more favourable change in the control arm than in the intervention arm

Analysis of ISH presentation rates alone returned almost identical rate ratios to the combined ED presentation/mortality data (Table 2, ISH presentations only row); while the intervention effect ratio for the mortality-only outcome has a slightly higher point estimate (intervention effect ratio = 1.18, 95% CI 0.5, 2.7) – though the small number of suicide deaths (Table 1) means that the precision of this estimate was too wide to be of material use (as anticipated).

## Discussion

The MISP-NZ study did not provide substantive evidence for the effectiveness of the intervention. This stands in contrast to the results of the Nuremberg, [9] Akita [3] and Hungary [22] multilevel intervention studies reporting a statistically significant reduction in suicidal behaviours. Part of this discrepancy might arise from differences in study design (discussed below under strengths and weaknesses) although other explanations are considered below.

### Strengths

The key strength of the MISP-NZ design was the randomisation of geographic regions to the control and intervention arms, in contrast to the Nuremberg, Akita and Hungary studies, where allocation was not randomised. This randomisation means the study has reduced risk of selection bias and improved comparability of intervention and control groups. This overcomes the weaknesses of the non-randomised studies where, in Akita and Nuremberg, there were very large baseline imbalances in the rates of suicide and suicidal behaviours as well as different socio-demographic profiles in the compared regions: for example, in the Nuremberg and Akita studies, regression to the mean in the intervention group could have accounted for a substantial portion of the observed difference between intervention and control sites during the trial. In light of this kind of challenge and the difficulty of matching in community trials with few clusters, the use of matched random assignment was advocated early on in the design of this study. Formal randomisation of regions enhances the internal validity of the MISP-NZ trial.

The second key strength is the accuracy of reports on ISH collected from emergency departments (ED) using note-review of a variety of electronic databases and clinical notes. The current accepted self-harm statistics in New Zealand are limited to individuals who were admitted to hospital for at least 48 h. Of note, is that the number of admissions to hospital is influenced by the availability of hospital beds, the risk tolerance of treating clinicians, and the treatment protocols of individual services (for instance mental health or ED). The data collection process was designed to minimise the impact of such variation by proactively investigating information documented in the ED, mental health and other hospital notes to maximise the detection of every relevant incident of ISH. The process included viewing general summary diagnoses and descriptions (for example, abdominal pain or wrist trauma) as flag indicators for continued search efforts (including probing multiple sets of clinical notes) to uncover information on suicidal/ISH behaviour or intent that was not evident at the summary level. Furthermore, the inclusion of instances of ‘probable self-harm’ and ‘suicidal ideation’ allowed for a sensitivity analysis (not reported here) to be performed revealing the addition of these further categories to the primary analysis made no difference to the overall findings. We can therefore be confident that our results have not been biased by misclassification of the ISH outcome.

We are also confident that misclassification in our self-inflicted death outcome is uncommon. Our initial investigation of the coronial data, which included active (suspected but not confirmed) and closed suicide cases, comparing the initial tentative cause of death and the final cause of death, showed that the majority of ‘self-inflicted’ cases in our analyses were ISH. Post hoc, our most recent review of the coronial data suggested that about 5.8% of “confirmed and suspected suicide” deaths were later classified as not suicide.

### Limitations

The key limitation of the MISP-NZ study was the short 25 month time frame for intervention implementation. Although a number of trials have reported an effect after 2 years of intervention (Nuremberg [9] and Hungary [22]), the time frame of the more recent multilevel intervention implemented in metropolitan and rural areas of Japan was extended to 3.5 years because no effect was evident after 2 years [23]. This suggests that 2 years of interventions may not always be enough to provide statistical power for comparison and/or to allow establishment and sufficient penetration of the intervention. Additionally, the baseline data collection period of 6 months was shorter than anticipated due to delays in contract negotiations with the funder. Ideally, a longer baseline is needed (Akita and Hungary studies both had a 3 year baseline, and Nuremberg had 1 year) to account for seasonality.

A second limitation, due to the population size, was the small absolute number of suicide deaths in each DHB region, and the relatively brief duration of the study. This meant that the study had to be designed to examine the combined death and self-harm presentation outcomes: the study was not powered to investigate the most relevant outcome of interest: self-inflicted death. This problem is not unique and it has been noted that it is often difficult to design a study with sufficient statistical power to detect an effect due to the low rate of suicide in the general population [23].

Another limitation is that cluster randomisation with a small number of clusters is more vulnerable to imbalance between study arms than individual randomisation, as there are fewer units of randomisation and therefore more correlated characteristics within members of clusters i.e. intra-cluster correlation. However, the primary analyses in this study report the estimates of rates, rate ratios, and the intervention effects using a summary-measures approach to dealing with the clustered nature of the data in addition to accounting for the matched component of the study design, so we do not think this affected our interpretation of the results.

Finally, the denominator data for each DHB used in the analysis was calculated from the 2006 New Zealand Census. This was due to the cancellation of the 2011 New Zealand Census following the Canterbury earthquake in February of that year, meaning that 2006 was the most recent census estimate available for calculation of the person-time denominators. If there had been substantially different population growth in the two study arms between 2006 and 2012 then the true estimate of the ratio of change in rate of ISH or self-inflicted deaths could be different than the estimate reported here.

### Possible explanations for our findings

A number of factors may explain our results. First, it is possible that the MISP-NZ interventions were not able to add enough extra value in addition to ‘practice as usual’ (PAU) to yield a large difference (i.e. the 20% reduction in rates on which sample size calculations were predicated). In New Zealand PAU consisted of significant national investment in mental health over several years prior to and during MISP-NZ. National depression awareness campaigns were well resourced and embedded nationally and thus not a separate component of the trial intervention package (which is the case for the EAAD based studies). Furthermore, work was already underway in the primary care setting to address mental health issues through better identification, referral pathways and treatment. Thus, it is possible that MISP-NZ, because it was designed and implemented considerably later than the other multilevel intervention trials, was delivered in a very different practice as usual context. We are unable to assess to what extent this is so, as no description of practice as usual has been provided in the previously published studies.

Second, MISP-NZ was not focussed specifically on the identification and treatment of depression in primary care, as the EAAD inspired trials are. This was due to the need for MISP-NZ interventions to be tailored to the existing national suicide prevention context which already included considerable investment in and work with primary care providers and the national depression awareness campaign. MISP-NZ interventions, although they included information and education and workshops on depression and other mental health issues, were more focused on the identification of suicide risk specifically (hence the use of QPR training). Also, arguably, the move away from a focus on depression identification and treatment was aligned to changes in thinking about suicide prevention that question the usefulness of the hitherto dominant explanatory model in suicidology where depression was positioned as the single most important risk factor for suicide. Pridmore, for example, notes that the evidence base underpinning the depression/mental illness link with suicide was faulty due to its reliance on the flawed design of psychosocial autopsy studies [40, 41]. Alternative thinking suggests that suicide prevention in future might be better to balance the mental illness and depression focus of interventions with the need also to consider non-health system risk factors found in wider social agency systems (justice, welfare and education for instance), and also to target interventions to specific population groups (men, elderly, LGBTI, ethnic groups) since each group has a different set of specific risk factors as suggested by the most recent systematic review of suicide prevention activities [42].

Third, the possibility of a degree of contamination cannot be excluded, where MISP-type interventions may have been implemented in control regions. The initial stocktakes of the control regions revealed some variation in the type and level of suicide prevention activities occurring at the start of the trial and the team were aware of some changes occurring during the 2 years, for instance, the Ministry of Health funded the appointment of suicide prevention coordinators in some regions. We were also alerted to some instances where community workers or professionals from the control regions were requesting and receiving the QPR training after hearing about its use in the intervention regions. Emergent local initiatives are more likely to occur when trials are run over longer periods, so should be accounted for in study design and planning phases.

Fourth, there are a number of reasons why the implementation of the interventions may not have been effective. A process evaluation following the MISP-NZ trial revealed that the uptake varied for the different intervention components, with the best uptake for print resources, followed by the suicide prevention training and workshops. Although engagement with the media was successful for promoting MISP-NZ activities and suicide prevention in general, attempts to engage the media in workshops on suicide reporting were generally unsuccessful. Perhaps most importantly, engagement with people working in primary care settings (nurses and GPs) was the most challenging aspect of the MISP-NZ project resulting in their limited engagement in the suicide prevention activities.

Finally, the health service, cultural and historical contexts of the countries where multilevel interventions have been tested are hugely varied. MISP-NZ is the first multilevel intervention for suicide prevention outside Europe and Japan. Cultural factors such as religion and the local civil context (for instance civil conflict and war) influence factors contributing to suicides, as do socio-cultural factors such as differences in gender role expectations. The diagnosis and treatment of mental illness (for instance prescribing patterns for antidepressants), considered alongside the contribution that mental illness makes to suicide risk, also varies greatly between countries [41]. This suggests that multilevel interventions for suicide prevention need to be tailored to country and context, and the depression focus of previous multilevel interventions may not be suitable for all settings. Further, we still do not understand whether some elements of the multilevel model of interventions are more effective than others, or if it is the synergistic effect of several or all components combined that are key to intervention success as the different levels of such interventions have not yet been evaluated in a controlled design [7].

### Implications for the future of multilevel interventions for suicide prevention

Although suicide is a statistically rare event, its social, economic and psychological impacts are so great that suicide prevention remains a priority. While multilevel interventions for suicide prevention have been positioned as the gold standard, they are hugely resource intensive and as such are likely only to be funded at the government level. This makes it probable that they will be tied to government policy and strategy which means study design is often shaped by externally imposed limits. To assist policymakers and to make good use of public funding, it is essential that the evidence base around the effectiveness of multilevel interventions for reducing suicidal behaviours is robust, scientific (more RCTs are required) [42] and reported transparently. While this study has followed the CONSORT guidelines for reporting of a cluster randomised controlled trial, we think there is a clear need, to assist in assessing the effectiveness of multilevel interventions and their external generalisability, for the development of reporting criteria for non-randomised controlled multilevel interventions to reduce suicidal behaviours. Based on our assessment of the published trials to date, we make a number of suggestions below.

#### Reporting criteria for multilevel interventions for suicide prevention

Descriptions of published multilevel intervention studies have frequently omitted important information, particularly contextual information about the local health system, and description of pre-existing suicide prevention activities (i.e. practice as usual).

Reporting should include a description of relevant country’s health system funding (public or private or mixed) due to the effects on patient access to assessment and treatment. As part of the health system context it would also be useful to note any significant temporal trends in suicide rates. Countries with high suicide rates at baseline have more opportunity to show an intervention effect than countries where suicide rates have already been reducing, such as in New Zealand. Similarly, if the intervention is to have a depression focus, it would be useful to report country level anti-depressant prescribing patterns at baseline.

Description of practice as usual should include documentation of any national suicide prevention strategy and its key components. This would include current or recent national media based public health campaigns and, if depression and its management is a key focus, existing GP awareness of depression and mental health problems, their diagnoses and treatment. It is also important to note existing or recent national investment in relevant features of the health system.

Other important aspects of study design that are often omitted include the decision or rationale for the designation of the control and intervention regions, and any stopping rules. In most of the studies, high suicide rates was cited as the reason for the selection of both the intervention and control regions. As regards stopping rules, we note that the most recent multilevel intervention trial in Japan [23] extended the intervention period after no effect was observed. It is possible that MISP-NZ may have produced an intervention effect if the study had been extended.

A further point deserving consideration is the extent to which multilevel interventions are expected to have impacts beyond the intervention period. Results from the Hungary study reported that the effects ceased post-intervention [22]. However, this may depend on the intervention mix as some components, such as de-stigmatisation campaigns, will be more likely to have longer term impacts than other components, for instance telephone help-lines that cease post-intervention (or in the case of MISP-NZ, the QPR training that ceased to be freely available). Other intervention components such as efforts to improve referral pathways may have mixed short and long term effects.

Finally, in some cases, depending on the intervention package and design, it may also be useful to report relevant economic, social or political characteristics of the country where the intervention is being implemented. For instance in countries experiencing high unemployment, reporting on the type of social welfare regimes will be important as these can buffer the financial burden of unemployment which is a risk factor for suicide.

## Conclusion

Although complex community interventions to address mental health or other health problems are increasingly used in many countries, they are a challenging to research robustly. In designing MISP-NZ we drew on knowledge generated from the public health and health services literature on complex trial methods to build on the Nuremberg, Akita Prefecture and EAAD evidence, and created a robust two-year multilevel community intervention tailored to the New Zealand setting. The results of this trial, while inconclusive, suggest that MISP-NZ had no effect on suicidal behaviours. This result is inconsistent with the results of similar multilevel intervention studies leading us to question the effectiveness of multilevel interventions for reducing suicidal behaviour and suggesting that a different approach may be required.
